# Neighbourhood Socioeconomic Deprivation and Older Adults’ Cognitive Decline in Porto, Portugal: A 13-Year (2005–2018) Longitudinal Analysis Using the Population-Based EPIPorto Cohort

**DOI:** 10.3389/ijph.2026.1608945

**Published:** 2026-04-15

**Authors:** Cláudia Jardim Santos, Carla Moreira, Ana Isabel Ribeiro

**Affiliations:** 1 EPIUnit ITR, Instituto de Saúde Pública da Universidade do Porto, Universidade do Porto, Rua das Taipas, Porto, Portugal; 2 Unidade de Investigação em Epidemiologia do Instituto de Saúde Pública da Universidade do Porto, Porto, Portugal; 3 Faculdade de Medicina da Universidade do Porto, Porto, Portugal; 4 Departamento de Medicina da Comunidade, Informação e Decisão em Saúde, Faculdade de Medicina da Universidade do Porto, Porto, Portugal; 5 Centro de Matemática da Universidade do Minho, Braga, Portugal; 6 Department of Geography, Faculdade de Letras da Universidade do Porto, Centre of Studies in Geography and Spatial Planning (CEGOT), Porto, Portugal

**Keywords:** age-friendly cities, cognitive decline, old age people, social determinants, urban health

## Abstract

**Objectives:**

Living in socioeconomically deprived areas has been linked to poorer health outcomes, with older adults potentially more vulnerable due to cumulative environmental exposure. This study examined the association between neighbourhood socioeconomic deprivation and cognitive decline among older adults in Porto, Portugal.

**Methods:**

We used data from 486 participants aged ≥50 years in the EPIPorto cohort, each with at least two cognitive assessments between 2005 and 2018. Neighbourhood deprivation was measured using the Portuguese European Deprivation Index; cognitive function was assessed with the Mini-Mental State Examination. Missing data were addressed using multivariate imputation (mice package), and associations were estimated via linear mixed-effects models (lme4 package).

**Results:**

The average cognitive decline was −0.60 points between assessments (95% CI: −0.82 to −0.37). In unadjusted models, higher neighbourhood deprivation was associated with faster decline (β = −0.18; 95% CI: −0.29 to −0.06), but this was not significant after adjustment (β = 0.00; 95% CI: −0.11–0.12). Greater decline was significantly associated with older age, female sex, and lower education.

**Conclusion:**

Findings highlight the role of individual sociodemographic factors but indicate no significant association with neighbourhood deprivation.

## Introduction

An increasing number of studies have shown that residing in deprived neighbourhoods is associated with increased mortality and ill health even after accounting for personal socioeconomic factors [1]. This association happens because neighbourhood deprivation is a marker for characteristics that can affect health, including the availability of public services and environmental resources, and therefore it can exert an independent effect on an individual’s health [1]. A growing body of research suggests that living in the most deprived neighbourhoods has been associated with poorer health outcomes, such as higher allostatic load [2], worse physical health-related quality of life [3], increased risk of multimorbidity [4], increased likelihood of polypharmacy, physical dependency, and previous acute hospital admission [5]. For older adults, living in deprived neighbourhoods is particularly relevant as they tend to spend most of their time in their residential neighbourhood and often face heightened vulnerability to its conditions due to long-term residence [6].

The ageing process includes natural neurobiological changes that can impair cognitive functions, including conceptual reasoning, processing speed, and memory decline. For instance, around 40% of people 65 years or older experience some form of memory loss [7], and 25% of adults aged 60 and older perceive noticeable declines in cognitive abilities [8]. The loss of cognitive capacities, particularly in individuals experiencing accelerated decline, can impair independent daily functioning [7], leading to difficulties with telephone and transportation use, medication management, and financial responsibilities [9]. Age-related cognitive changes, which typically occur without significant impact on daily functioning, differ from pathological cognitive decline, which can lead to disability, dependency, impaired communication, and reduced quality of life, making the latter a public health priority in the context of an ageing population [10, 11].

Several studies have found that the place where we live influences cognitive health [12–14]. The literature has found that middle-aged and older individuals who live in deprived neighbourhoods have lower cognitive performance [12, 13], even if they do not face personal socioeconomic disadvantages [14]. For instance, when analysing the life-course neighbourhood deprivation effect on the brain, people who live in disadvantaged neighbourhoods for most of their lives show greater structural impairments in the brain, such as alterations in specific brain areas which are connected to differences in cognitive performance in adulthood [15]. Moreover, the effect on brain morphology was most consistently observed when examining deprivation during mid-to-late adulthood [15]. Another study examined the influence of neighbourhood physical and social environments on cognitive function trajectories over 18 years. The findings indicated that, initially, these resources were not associated with cognitive function levels. However, over time, they were linked to cognitive health [16].

In 2019, Portugal had the fourth-highest old-age dependency ratio, with 38 persons aged 65 and older per 100 working-age individuals (20–64 years old) [17]. This trend is expected to continue, with Portugal remaining among the top ten countries or areas with the highest old-age dependency ratio by 2050 [17]. In 2023, nearly a third of Porto’s population was 60 years or older [18]. As the population ages, it is crucial to understand how the neighbourhood environment affects cognitive function, particularly in urban areas where most older adults live [19]. Furthermore, no studies in Southwest Europe have utilised longitudinal analyses to address this topic. Therefore, this longitudinal study examines the association between neighbourhood socioeconomic deprivation and older adults’ cognitive decline in community-dwelling older adults living in Porto.

## Methods

### Study Design and Participants

This study used data from the EPIPorto cohort, which ran its baseline assessment from 1999 to 2003, enrolling 2485 non-institutionalised adults from Porto, Portugal, through digit-dialling landline telephone numbers randomly selected [20]. To date, the EPIPorto cohort study has conducted five follow-up evaluations: follow-up 1 (2005–2008, n = 1,681), follow-up 2 (2013–2015, n = 995), follow-up 3 (2017–2018, n = 964), follow-up 4 (2020, n = 869), and follow-up 5 (2021–2022, n = 836). At each evaluation, trained interviewers conducted face-to-face interviews using a structured questionnaire to collect information on participants’ socioeconomic, behavioural, and clinical characteristics.

In the present study, we selected individuals 50 years or above at baseline. This age limit was established because the biological effects of age-related cognitive decline are more likely to be manifest from midlife onward; thus, it is likely that cognitive change over time can be detected [21]. Among these individuals, we further selected those who had at least two Mini-Mental State Examination (MMSE) assessments, as this was the minimum requirement to estimate within-person change in cognitive performance over time. To address missing MMSE data, we applied multiple imputation, which is most effective under the assumption that data are Missing at Random (MAR) [22]. We restricted our analysis to follow-up waves where less than 50% of MMSE data were missing, since multiple imputation performs best when a substantial proportion of data is observed and the missingness is not excessive [23]. The baseline, fourth, and fifth follow-ups had more than 50% missing MMSE data, which could compromise the reliability of the imputed estimates. Thus, we focused on data from the first, second, and third follow-up waves, which together span a 13-year period. This allowed for optimal use of the available information through multiple imputation.

Participants differed from non-participants in several respects. Non-participants were individuals who were aged 50 at baseline but did not have at least two Mini-Mental State Examination (MMSE) assessments. Compared to participants, non-participants were significantly older, had lower educational attainment, were more likely to have held manual occupations, and had lower MMSE scores. See Supplementary Material S1 for the full comparison.

### Neighbourhood Socioeconomic Deprivation

The European Deprivation Index for Portugal (EDI-PT) was used to measure neighbourhood socioeconomic deprivation in each evaluation. The EDI-PT categorises census block groups into quintiles (Q1-least deprived to Q5-most deprived) using individual-level data from the European survey on deprivation - the European Union-Statistics on Income and Living Conditions (European Union Statistics on Income and Living Conditions EU-SILC), and ecological data on deprivation from the Portuguese Census. We used the EDI-PT 2011, an ecological deprivation index that includes information on eight variables, namely, home ownership (renter, owner, other); presence/absence of indoor toilet flushing in the household; rooms in the household (≤5 rooms or ≥6 rooms); occupation class of the residents (lower white-collar, upper white-collar, and blue-collar workers); education level of the residents (primary, secondary or tertiary); employment condition (employee or employer); employment status (unemployed and looking for a job, or employed); and nationality (Portuguese or foreign) [24]. Because the EDI-PT uses census data, the occupation class reflects the distribution of occupations among economically active residents in each area, rather than the employment or retirement status of individual participants at the time of cohort participation. In this paper, the quintiles were treated as a continuous variable to capture the trend of deprivation across areas. This approach has also been applied in other research, such as in the study by Zelenina [25].

### Cognitive Function

Cognitive function was evaluated with the Portuguese version of the Mini-Mental State Examination (MMSE). This 30-item screening test assesses various cognitive domains: orientation, registration, attention and calculation, recall, and language. The MMSE is the most widely used short screening tool for assessing cognitive impairment for its reliability and validity [26]. The test yields a maximum score of 30 points, with each item worth one point. Higher scores indicate better cognitive function.

### Covariates

At each evaluation, participants provided self-reported data on their sociodemographic background. Variables included sex (female or male), date of birth (used to calculate age), marital status (dichotomised as partnered or non-partnered), and level of education. Educational attainment was categorised into four groups: less than primary education (fewer than four completed years), primary education (four to six years), secondary education (nine to twelve years), and tertiary education (more than twelve years, including university degrees). Current professional status was also recorded and classified as not in the workforce or, for those employed, as manual or non-manual workers. This classification was based on the Portuguese Classification of Occupations 2010, aligned with the International Standard Classification of Occupations (ISCO-08), with groups 0–5 considered non-manual and groups 6–9 classified as manual. Similar categorisation schemes of the covariates were employed in a previous study by the team [27]. To assess potential multicollinearity among covariates, we calculated variance inflation factors (VIFs) for the fully adjusted linear regression model. All VIFs were below 2, indicating no evidence of problematic collinearity.

### Statistical Analysis

Continuous variables were summarised using means and standard deviations, while categorical variables were described using absolute frequencies and corresponding percentages.

Both crude and adjusted regression models were used to evaluate the association between neighbourhood deprivation (exposure variable) and MMSE scores at each follow-up (outcome variable), adopting a cross-sectional perspective. Fully adjusted models included potential confounders: age, sex, marital status, educational attainment, and current professional activity.

In addition to cross-sectional analyses, a longitudinal analysis was performed to assess the association between neighbourhood socioeconomic deprivation and cognitive performance over time.

To model the trajectory of MMSE scores over time while accounting for variability at both individual and contextual levels, we fitted a Linear Mixed-Effects Model (LMM) using restricted maximum likelihood estimation (REML), via the lme4 R package (version 1.1.35.5). We calculated 95% confidence intervals (CI) for all estimated coefficients. These coefficients represent the adjusted association between each covariate and the MMSE score. For continuous variables, coefficients reflect the average change in MMSE score per unit increase in the covariate. For categorical variables, they represent the mean difference in MMSE score relative to the reference group.

The model was mathematically specified as follows:

$${MMSE}_{ijk}=\beta 0+\beta 1\times {time}_{ijk}+\beta 2\times {Neighbourhodd\;deprivation}_{j}+\beta 3\times {Age}_{i}+\beta 4\times {Sex}_{i}+\beta 5\times {Marital\;Status}_{i}+\beta 6\times {Educational\;attainment}_{i}+\beta 7\times {Professional\;activity\;}_{i}+{b0}_{j}+{b1}_{j}\times {time}_{ijk}+{u0}_{i}+{u1}_{i}\times {time}_{ijk}+\varepsilon_{ijk}$$

Where:

- i index the individual,
- j indexes the contextual group (Neighbourhood deprivation quintile),
- k indexes the repeated measurements over time,
- β are the fixed-effect coefficients,
- b0_j_ and b1_j_ are the random intercept and slope for time at the contextual level (neighbourhood deprivation), assumed to follow a multivariate normal distribution N(0, Ψ)
- u0_i_ and u1_i_ are the individual-level random intercept and slope for time, assumed N(0, Σ),
- ε_ijk_∼N(0,σ^2^) is the residual error term.

This model structure allows for both between-individual and between-context heterogeneity in baseline cognitive performance and its change over time, making it particularly suited for longitudinal data with hierarchical structure.

Model assumptions, including normality of residuals and homoscedasticity, were assessed through graphical inspection of standardised residuals and fitted values.

All analyses were conducted following multiple imputation of missing values in cardiovascular-related measures and adjustment for covariates. Assuming the missing at random (MAR) mechanism, which our data suggest is plausible, we applied Multivariate Imputation by Chained Equations (MICE) using fully conditional specification via the mice R package (version 3.14.0). The results were pooled using Rubin’s rules.

All the analyses were conducted using the R statistical software, version 4.2.1. Statistical significance was considered at p-value <0.05.

We also conducted a sensitivity analysis to assess the robustness of our findings by focusing on individuals who did not move house during the study period to control for potential changes in neighbourhood deprivation, as residential mobility could introduce variability in exposure to neighbourhood characteristics and, therefore, influence cognitive decline (Supplementary Material S2. The results of this analysis were consistent with the main findings, suggesting that residential mobility did not significantly affect the observed associations.

## Results

The dataset included 486 individuals, 303 females (62.3%) and 183 males (37.7%), who had at least two Mini-Mental State Examination assessments during the first, second, or third follow-ups between 2005 and 2018. Table 1 presents the sociodemographic characteristics of the participants, after imputations; the corresponding table with characteristics before imputation is provided in Supplementary Material S3. As expected, the cohort’s mean age increased from 65.8 years (standard deviation [SD] = 7.0) in the first follow-up to 73.5 years (SD = 7.0) in the second, and 77.4 years (SD = 7.1) in the third. Throughout all evaluations, more than half of the participants were partnered, and primary education was the most frequently reported level of education. Regarding professional activity, most participants worked in non-manual professions during the first evaluation. As expected, by the second and third follow-ups, almost everyone was no longer in the workforce. For the Mini-Mental State Examination, the average score was 28.1 points (SD = 1.9) in the first, in the second follow-up, the average score was 27.7 points (SD = 2.2), and in the third follow-up, the average score was 26.9 points (SD = 2.4).

**TABLE 1:** Descriptive characteristics, after multiple imputations, of the EPIPorto participants included in the study (n = 486, 303 females; 183 males). Porto, Portugal, 2005-2018.

Variables	Counts (%) or mean (standard deviation)		
	1st follow-up (2005–2008)	2nd follow-up (2013–2015)	3rd follow-up (2017–2018)
Age (years)	65.8 (7.0)	73.6 (7.0)	77.4 (7.1)
Marital status			
Partnered	336 (69.1%)	309 (63.6%)	274 (56.4%)
Non-partnered	150 (30.9%)	177 (36.4%)	212 (43.6%)
Education			
Lower than primary	44 (9.1%)	46 (9.5%)	47 (9.7%)
Primary	220 (45.3%)	234 (48.1%)	201 (41.4%)
Secondary	125 (25.7%)	109 (22.4%)	143 (29.4%)
Tertiary	97 (20.0%)	97 (20.0%)	95 (19.5%)
Professional activity			
Manual	47 (9.7%)	10 (2.1%)	7 (1.4%)
Non-manual	385 (79.2%)	26 (5.3%)	4 (0.8%)
Not in the workforce	54 (11.1%)	450 (92.6%)	475 (97.7%)
Neighbourhood socioeconomic deprivation			
Quintile 1 (least deprived)	114 (23.5%)	108 (22.2%)	108 (22.2%)
Quintile 2	110 (22.6%)	114 (23.5%)	116 (23.9%)
Quintile 3	83 (17.1%)	84 (17.3%)	86 (17.7%)
Quintile 4	84 (17.3%)	88 (18.1%)	88 (18.1%)
Quintile 5 (most deprived)	95 (19.5%)	92 (18.9%)	88 (18.1%)
Mini-mental test examination	28.1 (1.9)	27.7 (2.2)	26.9 (2.4)

Table 2 presents the results of the cross-sectional analyses examining the associations between neighbourhood deprivation and the MMSE at each follow-up. The crude analyses revealed that living in more deprived neighbourhoods was associated with significantly lower MMSE scores: −0.23 (95% CI: −0.35, −0.12) at the first follow-up; −0.18 (95% CI: −0.32, −0.04) at the second; and −0.25 (95% CI: −0.40, −0.10) at the third. However, after adjusting for potential confounders, including age, sex, marital status, education, and profession, these associations were no longer statistically significant.

**TABLE 2:** Cross-sectional analysis of the association between Neighbourhood socioeconomic deprivation quintiles and the Mini-Mental Examination (MMSE). Porto, Portugal, 2005-2018.

Variables	Crude		Adjusted^a^	
	*β*	95% CI	*β*	95% CI
MMSE at 1st follow-up	**−0.23**	**(**−**0.35,** −**0.12)**	0.01	(−0.09, 0.11)
MMSE at 2nd follow-up	**−0.18**	**(**−**0.32,** −**0.04)**	0.02	(−0.09, 0.14)
MMSE at 3rd follow-up	**−0.25**	**(**−**0.40,** −**0.10)**	−0.06	(−0.20, 0.07)

^a^ Adjusted for age, sex (female/male), marital status (partnered/non-partnered), education (lower than primary, primary, secondary, and tertiary education), and profession (not in the workforce, manual, non-manual). Bold indicates statistically significant results.

Table 3 shows the estimated rates of cognitive decline across follow-ups according to neighbourhood socioeconomic deprivation. The average rate of cognitive decline among the cohort was −0.60 points between consecutive assessments (95% Confidence Interval [CI]: −0.82 to −0.37).

**TABLE 3:** Rates of cognitive decline (n = 486) across follow-ups according to neighbourhood socioeconomic deprivation and covariates, per follow-up evaluation. Porto, Portugal, 2005-2018.

Variables	Rate of cognitive decline β (95% CI)
	Crude model
Time per follow-up evaluation	**−0.60 (-0.82, -0.37)**
Neighbourhood socioeconomic deprivation	**−0.18 (-0.29, -0.06)**
​	Adjusted model^a^
Time	−**0.52 (-0.71, -0.33)**
Neighbourhood socioeconomic deprivation	0.00 (−0.11, 0.12)
Age	**−0.05 (-0.07, -0.03)**
Sex	
Male	Reference category (Ref.)
Female	**−0.52 (-0.78, -0.33)**
Marital status	
Partnered	Ref.
Non-partnered	0.06 (−0.17, 0.29)
Education	
Tertiary	Ref.
Secondary	−0.25 (−0.56, 0.05)
Primary	**−1.24 (-1.54, -0.94)**
Lower than primary	**−3.15 (-3.57, -2.73)**
Profession	
Non-manual	Ref.
Manual	0.04 (−0.37, 0.45)
Not in the workforce	**0.56 (0.28, 0.80)**
Interaction term	
Neighbourhood socioeconomic deprivation x sex (ref. Male)	−0.05 (−0.22, 0.10)
Neighbourhood socioeconomic deprivation x manual (ref. non-manual)	−0.05 (−0.34, 0.23)
Neighbourhood socioeconomic deprivation x not in the workforce (ref. non-manual)	−0.02 (−0.17, 0.13)

^a^ Adjusted for age, sex (female/male), marital status (partnered/non-partnered), education (lower than primary, primary, secondary, and tertiary education), and current profession (not in the workforce, manual, non-manual). Bold indicates statistically significant results.

When examining the association between neighbourhood socioeconomic deprivation and cognitive decline, we found that each quintile increase in neighbourhood deprivation was associated with an average rate of cognitive decline of −0.18 (95% CI: −0.29 to −0.06) between consecutive assessments. However, when the model was adjusted, this association was no longer statistically significant (*β* = 0.00, 95% CI: −0.11–0.12).

The results also show that older age (β: −0.05; 95% CI: (−0.07, −0.03)), being female (β: -0.52; 95% CI: (−0.78, −0.33)), and lower educational attainment (β for less than primary education: -3.15; 95% CI: -3.57 to −2.73; β for primary education: -1.24; 95% CI: −1.54 to −0.94) are significantly associated with cognitive decline. Not being in the workforce (β: 0.56; 95% CI: 0.28, 0.80) was significantly associated with cognitive improvement.

Lastly, no significant interaction effects were found between neighbourhood socioeconomic deprivation and sex or occupational status, indicating that the impact of deprivation on cognitive function appears to be consistent across these demographic and socioeconomic groups.

## Discussion

Our study aimed to investigate the association between neighbourhood socioeconomic deprivation and older adults’ cognitive decline in community-dwelling older adults living in Porto over a 13-year follow-up period. We found that, over time, older adults experience cognitive decline, with a greater decline observed among those living in more deprived neighbourhoods. However, this association was no longer significant after adjusting for age, sex, marital status, education, and current profession. Additionally, older age, being female, and lower educational attainment remained significantly associated with greater cognitive decline over time.

Our findings indicate that, with time, individuals’ cognitive abilities tend to decline. This is in line with what is consistently described in the literature. With ageing, neurobiological changes occur in the brain, including synaptic loss, which contributes to cognitive deterioration. However, these changes differ from the mechanisms seen in neurological diseases such as Alzheimer’s disease, where brain volume loss results from both neuronal and synaptic loss [8]. So, experiencing a decline in cognitive abilities over time is a natural part of the ageing process. Thus, it was with no surprise that we also found that the risk of cognitive impairment increases with time. This highlights the importance of identifying factors that can help slow the decline in cognitive abilities, enabling older adults to maintain autonomy for longer. Preserving cognitive function in older age is crucial for daily activities such as taking medication correctly, driving safely, and managing household finances [12]. However, according to Hensel et al., a change of −0.18 points in the MMSE per evaluation is within the expected variability and may not indicate a clinically meaningful decline [28].

In our study, we found that individuals living in more deprived neighbourhoods had a greater cognitive decline. However, after adjusting for age, sex, marital status, education, and current profession, this association was no longer statistically significant [13, 29]. This was unexpected, as previous longitudinal studies have shown that poorer urban environment characteristics, which are often found in deprived neighbourhoods, are typically associated with worse cognitive health. Moreover, living in disadvantaged areas during mid-to-late adulthood was linked to lower cognitive ability and a faster decline in cognitive function over time [13]. In those studies, the sample sizes were considerably larger (1,160 [29] and 1,091 participants [13]), whereas our analysis included 486 individuals. Although the direction of the association between neighbourhood deprivation and cognitive performance was as expected, the lack of statistical significance needs further consideration. Beyond sample size limitations, several contextual factors may help explain this result. In the Portuguese context, socioeconomically deprived neighbourhoods may not display the same intensity or spatial concentration of environmental injustice or segregation as observed in other European settings, partly due to historical patterns of urban development [30] and comparatively weaker spatial polarisation [31]. This may, in turn, attenuate the health effects of area-level deprivation. Evidence suggests that in socioeconomically deprived areas, communities may develop stronger informal support systems and neighbourhood cohesion, which can buffer against adverse health outcomes [32]. In the same EPIPorto cohort used in the present study, a four-year prospective analysis found that older adults with stronger social support were significantly less likely to experience cognitive impairment [32]. Although not focused on cognitive health specifically, findings from a population-based longitudinal study showed that higher levels of neighbourhood social cohesion mitigated the adverse mental health effects associated with living in deprived areas [33]. These potential protective factors should be explored in future research. However, individual-level factors such as sex, marital status, level of education, and current profession remained significantly associated with cognitive function. These findings align with research showing that having a higher degree of education, even after adjusting for family background and genetics, is related to better cognitive health in later life [34]. This relationship can be explained by the cognitive reserve hypothesis, which suggests that engaging in intellectually stimulating activities enhances cognitive resilience, enabling individuals to better cope with age-related decline or neurological conditions [35]. However, it was surprising that individuals no longer in the workforce showed an improvement in cognitive health compared to those in non-manual professions, which are typically more cognitively demanding and would be expected to offer some protection against cognitive decline. This finding should be interpreted cautiously and may reflect selection effects rather than a true protective effect of labour market exit. Individuals who remain cognitively healthier may be more likely to participate in follow-up assessments after retirement, while those experiencing cognitive or health decline may be less likely to return. In addition, retirement may reduce exposure to work-related stress and time constraints, potentially allowing greater engagement in health-promoting or cognitively stimulating activities. Additionally, we observed that individuals in manual occupations experienced a greater decline in cognitive function compared to those in non-manual occupations. For example, a study on occupation-related differences in cognitive ageing found that professional occupations (requiring a university degree) and higher-skilled workers generally demonstrated better baseline cognitive ability and more favourable age-related retention, with academics (teaching and research professionals) performing best overall [36]. In contrast, the most pronounced cognitive declines were observed in two skilled trades groups—construction/building and textile/printing—both of which are manual occupations [36]. It should be noted that the predominance of non-manual occupations in this cohort may reflect selective participation, potentially under-representing individuals from lower socio-economic backgrounds, among whom manual occupations are more common. This may limit the ability to fully capture occupational inequalities in cognitive ageing. In our study, being female was linked to higher cognitive decline compared to men. This may be partly explained by biological factors, such as oestrogen levels. Following menopause, oestrogen levels stabilise at chronically low concentrations, diminishing the hormone’s neuroprotective effects on the cholinergic system (which plays an important role in various aspects of cognitive function), which may, in turn, contribute to cognitive ageing in women [36]. Moreover, women tend to have lower grey matter volume, which may make them more susceptible to the accelerated loss of grey matter associated with ageing [36].

### Strengths, Limitations and Further Studies

This longitudinal study followed participants for 13 years during a life stage when cognitive decline is more pronounced, making it well-suited for assessing changes in cognitive function over time. Moreover, this type of study allowed for a more accurate understanding of the sequence of events, which is important for assessing causation. This use of repeated measures is useful to detect subtle cognitive changes as time goes on. Additionally, collecting data at multiple time points helped reduce recall bias, as participants reported on their cognitive capacities in real-time. The study also utilised the Mini-Mental State Examination (MMSE), a widely used tool for assessing overall cognitive performance, which has been shown to correlate with atrophy in specific brain regions within the limbic system [8]. Since the limbic system plays a key role in emotion formation and processing, learning, and memory, its deterioration is particularly relevant to cognitive decline. Furthermore, by using multiple imputations, we addressed missing data, which is a common issue in longitudinal studies due to participant withdrawal or loss to follow-up. This approach helps preserve information, improve precision, maintain statistical power, and minimise the risk of biased estimates, ultimately strengthening the validity of our findings [37]. However, our study has some limitations. Since it relies on data from a closed cohort, there is a risk of selection bias, as individuals who remain in the study may differ from those who drop out, for example, potentially being healthier, leading to an underestimation of cognitive decline. Additionally, we observed only minimal changes in Mini-Mental State Examination (MMSE) scores over time, with a slight increase of 0.2 points between the first and second follow-up. This could reflect a healthy survivor effect, whereby individuals with better cognitive function are more likely to remain in the study over time. Moreover, a comparison between the analytical sample (participants) and those excluded from the analysis (non-participants) revealed that participants tended to live in less deprived neighbourhoods, had higher educational attainment, and were more likely to have held non-manual occupations. These differences suggest that the analytical sample may represent a healthier and more socioeconomically advantaged subgroup of the original cohort (see Supplementary Material S1). This potential selection process may partially explain the limited variation in cognitive decline observed. In this context, the largely stable cognitive scores and the absence of more pronounced neighbourhood-level associations should be interpreted with caution, as they may reflect attenuation due to selective survival and differential follow-up participation rather than a true absence of neighbourhood influence. Such processes are widely recognised in longitudinal ageing studies, where socio-economic inequalities in mortality and retention may limit the ability to fully capture the socio-economic gradient in cognitive ageing [38]. Additionally, the large share of missing cognitive data at the fourth and fifth follow-up waves should be taken into account. Cognitive data missing at later follow-ups are unlikely to be missing completely at random, because people with lower cognitive performance, poorer health, living in an institution are less likely to return for repeated assessments, alongside participant loss due to mortality over time. People whose cognitive abilities decline more quickly may be less likely to remain in the study, so they may show up less often in later waves. Because the missing data were not random, the cognitive trends may look more stable than they really were, so the results should be read with care. In our study, MMSE scores were analysed as continuous variables without applying education-specific cut-offs. Future studies could consider using education-adjusted MMSE thresholds to facilitate comparisons and more accurately capture cognitive impairment relative to educational attainment. Furthermore, from a life-course perspective, mid-life represents a critical period for brain health, during which modifiable risk and protective factors may have long-term implications for cognitive ageing. Public health recommendations increasingly emphasise mid-life interventions targeting cardiovascular health, physical activity, cognitive engagement, and environmental exposures as strategies to promote cognitive resilience later in life [39]. Cognitive function represents one dimension of health in later life, and unmeasured aspects of frailty may influence observed cognitive trajectories [40]. Future studies examining cognitive reserve and decline would benefit from incorporating multidimensional frailty measures to better capture overall health status and its interaction with cognitive ageing. While our findings suggest that individual-level factors play a crucial role in cognitive health, future research should explore other pathways through which neighbourhood environments may still contribute to cognitive decline. This could include examining the cumulative effects of long-term exposure to neighbourhood environmental stressors, such as air pollutants relevant to cognitive health (e.g., fine particulate matter), climatic factors like the urban heat island effect, the built environment (e.g., walkability), and proximity to urban green spaces.
