# Supplementary material for: Neighbourhood Socioeconomic Deprivation and Older Adults’ Cognitive Decline in Porto, Portugal: A 13-Year (2005–2018) Longitudinal Analysis Using the Population-Based EPIPorto Cohort
Source: Int J Public Health. 2026 Apr 15;71:1608945. doi: 10.3389/ijph.2026.1608945 (PMC13124638; doi:10.3389/ijph.2026.1608945)
Supplement: Supplementary file 1 [file DataSheet1.pdf]

Supplementary material

S1: Descriptive characteristics of EPIPorto participants eligible for inclusion in the analysis (aged ≥50 years at baseline and with data from at least two MMSE assessments - participants), in comparison with those who did not fulfil these criteria (non-participants).

	Participants (n=486)	Non-participants (n=971)	p-value
Mean age (range)	60.4 (50-80)	65.9 (50-92)	<0.001
Sex			
Female	303 (62.3%)	591 (60.9%)	0.624
Male	183 (37.7%)	380 (39.1%)	
Marital Status			
Partnered	361 (74.3%)	651 (67.0%)	0.006
Non-partnered	125 (25.7%)	319 (32.9%)	
Missings	0 (0.0%)	1 (0.1%)	
Education			
Lower than primary	45 (9.3%)	185 (19.1%)	<0.001
Primary	229 (47.1%)	506 (52.1%)	
Secondary	114 (23.5%)	172 (17.7%)	
Tertiary	97 (20.0%)	106 (10.9%)	
Missing	1 (0.2%)	2 (0.2%)	
Professional activity			
Manual	60 (12.3%)	186 (19.1%)	<0.001
Non-manual	385 (79.2%)	663 (68.3%)	
Not in the workforce	41 (8.4%)	120 (12.4%)	
Missings	0 (0.0%)	2 (0.2%)	
Neighbourhood socioeconomic deprivation			
Quintile 1	115 (23.7%)	177 (18.2%)	0.002
Quintile 2	113 (23.3%)	178 (18.3%)	
Quintile 3	78 (16.4%)	213 (21.9%)	
Quintile 4	85 (17.5%)	206 (21.2%)	
Quintile 5	95 (19.5%)	196 (20.2%)	
Missings	0 (0.0%)	1 (0.1%)	
Mini-Mental Test Examination	27.8 (2.5)	26.1 (3.7)	<0.001
Missings	379 (77.9%)	472 (48.6%)	

*p-values were calculated using the Wilcoxon test (for continuous variables) and the chi-square test (for categorical variables)

S2: Sensitivity analysis of people who did not move houses during the analysis period, per follow-up evaluation

	**Rate of cognitive decline (95% CI)**
	**Crude model**
**Time** per follow-up evaluation	**-0.59 (-0.76, -0.35)**
**Neighbourhood socioeconomic deprivation**	**-0.18 (-0.30, -0.06)**
	**Adjusted model[a]**
**Time**	**-0.53 (-0.74, -0.32)**
**Neighbourhood socioeconomic deprivation**	-0.00 (-0.11, 0.11)
**Age**	**-0.05 (-0.07, -0.04)**
**Sex**	
Male	Reference category (Ref.)
Female	**-0.50 (-0.77, -0.23)**
**Marital status**	
Partnered	Ref.
Non-partnered	0.03 (-0.20, 0.26)
**Education**	
Tertiary	Ref.
Secondary	-0.24 (-0.55, 0.07)
Primary	**-1.22 (-1.53, -0.92)**
Lower than primary	**-3.18 (-3.61, -2.75)**
**Profession**	
Non-manual	Ref.
Manual	-0.02 (-0.44, 0.40)
Not in the workforce	**0.54 (0.27, 0.81)**

S3: Descriptive characteristics, before multiple imputations, of the EPIPorto participants who met the selection criteria: were at least 50 years old at baseline, and had data on at least two Mini-Mental State Examination (MMSE) assessments at the first, second, and third follow-ups (n = 486, 303 females; 183 males).

**Variables**	**Counts (%) or mean (standard deviation)**		
	1st follow-up (2005-2008)	2nd follow-up (2013-2015)	3rd follow-up (2017-2018)
Age (years)	65.8 (7.0)	72.9 (6.9)	76.4 (6.8)
Marital Status			
Partnered	332 (68.3%)	279 (57.4%)	242 (49.8%)
Non-partnered	146 (30.0%)	147 (30.2%)	175 (36.0%)
Missing	8 (1.6%)	60 (12.3%)	69 (14.2%)
Education			
Lower than primary	43 (8.8%)	35 (7.2%)	36 (7.4%)
Primary	220 (45.3%)	199 (40.9%)	166 (34.2%)
Secondary	124 (25.5%)	104 (21.4%)	125 (25.7%)
Tertiary	91 (18.7%)	87 (17.9%)	89 (18.3%)
Missing	8 (1.6%)	61 (12.6%)	70 (14.4%)
Professional activity			
Manual	47 (9.7%)	3 (0.6%)	5 (1.0%)
Non-manual	379 (78.0%)	18 (3.7%)	4 (0.8%)
Not in the workforce	52 (10.7%)	404 (83.1%)	408 (84.0%)
Missings	8 (1.6%)	61 (12.6%)	69 (14.2%)
Neighbourhood socioeconomic deprivation			
Quintile 1 (least deprived)	114 (23.5%)	108 (22.2%)	108 (22.2%)
Quintile 2	110 (22.6%)	114 (23.5%)	116 (23.9%)
Quintile 3	83 (17.1%)	84 (17.3%)	86 (17.7%)
Quintile 4	84 (17.3%)	88 (18.1%)	88 (18.1%)
Quintile 5 (most deprived)	95 (19.5%)	92 (18.9%)	88 (18.1%)
Mini-Mental Test Examination	27.6 (2.1)	27.8 (2.1)	27.1 (2.4)
Missings	233 (47.9%)	61 (12.6%)	69 (14.2%)
